# Soil management is key to maintaining soil moisture in urban gardens facing changing climatic conditions

**DOI:** 10.1038/s41598-018-35731-7

**Published:** 2018-12-03

**Authors:** Brenda B. Lin, Monika H. Egerer, Heidi Liere, Shalene Jha, Stacy M. Philpott

**Affiliations:** 1 CSIRO Land and Water Flagship, 117-121 Station Street, PMB 1, Aspendale, VIC, 3195 Australia; 2 Environmental Studies Department, University of California, Santa Cruz, Santa Cruz, USA; 3 Environmental Studies Department, Seattle University, Seattle, USA; 4 Integrative Biology Department, University of Texas at Austin, Austin, USA

## Abstract

Urban gardens are vital green spaces, providing food for residents and space for engaged citizenry and community development. In California, climate change conditions (heat and drought) are becoming more extreme, threatening the resilience of urban gardens. Water use restrictions limit the timing and amount of water that gardeners can access, exacerbating these climate challenges for urban food production. Together with volunteer gardeners, we examined how ambient temperature, water use, vegetation, ground cover, and soil management affect rates of soil moisture gain and loss in urban gardens for a six-week period in the summer of 2017, during the hottest part of the growing season. We found that plot-level management of soils is essential for creating urban garden plots that maintain stable levels of water within garden soils. Although plots with better soil quality (i.e. water holding capacity) experienced slower rates of soil moisture gain after a watering event, they also experienced slower rates of soil moisture loss after the event, leading to soils with more stable, less fluctuating moisture profiles over time. This may benefit gardeners because under extreme climates (such as heat and drought) and water use restrictions, maintaining more stable soils for their plants means that the soils will retain water over a longer period after each watering event. Overall, such results highlight that better soil management that improves soil quality measures such as water holding capacity are potential solutions for maintaining soil moisture and reducing water use under changing climate conditions.

## Introduction

### Urban gardens are vulnerable to climatic change

Urban gardens are important sites of food production in cities, providing essential nutrients for food insecure communities and culturally appropriate foods where the traditional fruits and vegetables are unavailable in retail stores^1,2^. Urban gardens have been important sources of nourishment in cities for thousands of years, from the classic Mayan civilizations and Byzantine Constantinople^3^ to many current industrialized cities around the world^4,5^. Growing concerns about the quality, cost of food, and food insecurity in many cities around the world have increased interest in growing food locally through the development of urban community gardens^6,7^. Besides enhanced nutrition, gardens promote public health and improve quality of life by providing the space and opportunities to build social capital and community cohesion^8^.

However, extreme climate conditions such as extended periods of high heat or drought can severely affect the productivity and survivorship of food crops in cities^9,10^. Heat as well as water availability are essential considerations to local production, especially in urban gardens. Urban areas generally register 5–11 °C warmer than surrounding areas due to urban heat effects^11^, and gardens surrounded by more impervious land cover exhibit higher temperatures for longer periods than gardens surrounded by less urbanized areas with more natural vegetation^12,13^. Additionally, urban gardens are usually irrigated by public water systems that are dependent on the water availability of the catchment. In drought prone areas, water restrictions stemming from city or garden regulations can limit water input for the gardens. Water limitation is especially problematic for crop production during extreme temperature events when plants need water the most for evapotranspiration and cooling^14^. Such water stresses can lead to plants that suffer from high heat and sun scorch, making them more susceptible to other forms of fungal or pest damage that threaten plant survival^15,16^.

In California, USA, climatic changes have increased the frequency, magnitude, and duration of drought episodes that often co-occur with elevated temperature and heat waves^17,18^, and such climate impacts can have large ramifications for both rural and urban food production. Urban heat islands can further exacerbate the effects of warming climate conditions^19^. Due to this climate cycle and the lack of water storage during one of California’s worst droughts from 2012–2014^20^, water use reductions were mandated in California and are expected to become more extreme in the upcoming decades with increased forecasted fire and drought events^21^. This has led to stricter local regulations of water supply that also affect water availability for urban community gardens, with garden managers restricting water use amount and timing (for example, the San Jose Community Garden Program^22^).

### Can garden management factors help gardeners adapt to changing conditions?

**Figure 1:**
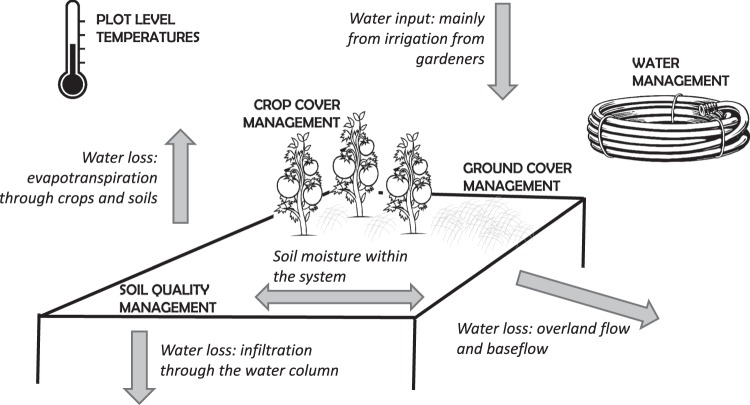
Water balance in community gardens follows many basic water balance conventions, but is constrained by the urban context of irrigation and water regulations as well as by individual gardener decisions made at the plot scale. Water balance patterns are described with gray arrows and in italicized text. Water balance is largely controlled by gardener management decisions for watering, crop management, soil management, and ground cover management. Water use was monitored by volunteer gardeners with a water gauge; in addition, climatic (temperature), crop cover, ground cover, and soil quality variables were collected. These measured variables are highlighted in the diagram using capitalized letters. Figure created by BBL. All clipart has been downloaded from Pixabay.com and used under a CC0 Creative Commons license (https://creativecommons.org/publicdomain/zero/1.0/deed.en). The clip art for each item can be found at: tomato plant (https://pixabay.com/en/tomatoes-vines-tomato-vegetables-40280/), thermometer (https://pixabay.com/en/cold-cool-hot-icon-measure-1293305/), garden hose (https://pixabay.com/en/garden-gardening-hose-pipe-water-2027548/).

Water balance principles in urban agriculture are similar to general water balance principles in any other type of ecosystem, with water primarily entering the system through rainfall and irrigation and water leaving the system through soil infiltration, surface runoff, baseflow, and evapotranspiration^23^. Soil moisture is considered a crucial link between hydrological and biogeochemical processes that synthesizes the interaction between climate, soil, and vegetation; thus, it is an essential water balance component to urban community gardens and the production of food^24^. Although a soil moisture deficit is not uncommon during seasonal droughts in California^20^, particular management decisions may affect the amount of water moisture that can be held in the system (Fig. 1 – a description of water balance factors provided in italicized font).

In the California central coast region, rainfall is severely limited during the summer months when most urban gardeners grow their crops. Because irrigation is the primary water input into gardens, watering decisions by gardeners essentially determine the amount and timing of water available for crops. Additionally, in urban community gardens, where plots are considerably smaller than in rural agriculture, individual plot owners also make specific decisions about their crops, ground cover, and soils, creating a range of mixed management at a small scale. These management practices can affect the water balance of the system by either ameliorating or exacerbating the larger climatic effects surrounding the garden.

Often times, urban gardens come from a mixed land use legacy, developing from vacant or abandoned land that might be contaminated or compacted^25^. Such conditions make it difficult to grow food crops directly in the soil, and a certain amount of preparation is required before crops can be successfully grown^26–28^. For example, some gardeners develop high quality soils that sit on top of otherwise unsuitable soils in order to grow their crops^29^. Gardeners make unique decisions about the way they manage their soils, often cultivating soils with their own mix of base material, compost, and other nutrient amendments^30^. This individuality makes garden plots ideal testbeds for examining distinct garden management impacts on water storage. Soil physical properties, such as texture, density, and organic matter content can all strongly affect soil water retention and water holding capacity^31^. Thus, soil management decisions by individual gardeners, including which materials and amendments they use, may play a large role in the efficient use of water within these urban garden systems.

For crops, water availability is essential to support plant metabolic processes and for evaporative cooling, which is especially important under high temperatures^32^, and it becomes more important to maintain water within urban gardening systems as temperatures increase. Vegetation management, such as increased shade cover from greater structural diversity, can reduce local ambient temperatures for crop plants and reduce soil surface temperatures at the plot level, lowering evapotranspiration rates and preserving water within the soil^9^. Indeed, research from plant fa**ç**ades have shown that different species can differentially reduce air and surface temperatures based on their transpiration potential and their leaf surface area and morphology^33^. This suggests that increasing coverage and structural diversity of crops within a plot could potentially improve soil moisture retention. Additionally, ground covers such as mulch and straw can protect soil surfaces, significantly reducing soil evaporation rates, increasing water use efficiency, and potentially increasing yields^34^.

In this study, we aimed to understand how the combination of individual decisions regarding watering, vegetation, ground cover, and soil management affect a gardener’s ability to maintain soil moisture within their plots during the summer growing months, when the combination of high heat and drought conditions are more extreme. We worked with twenty volunteer participant gardeners (n = 20 plots) across four urban community gardens in the Central Coast of California to document their water use for a six-week period, during the hottest part of the summer (August-September 2017). During this time, we also measured temperature, vegetation, ground cover, and soil parameters of their plots. We then used this information to better understand how gardener decisions are driving soil moisture within urban food production systems and to determine what management factors can help maintain higher soil moisture in gardens (Fig. 1 – a description of collected management information in capitalized font).

## Results

### Pattern of soil moisture gain and loss within urban gardens

**Figure 2:**
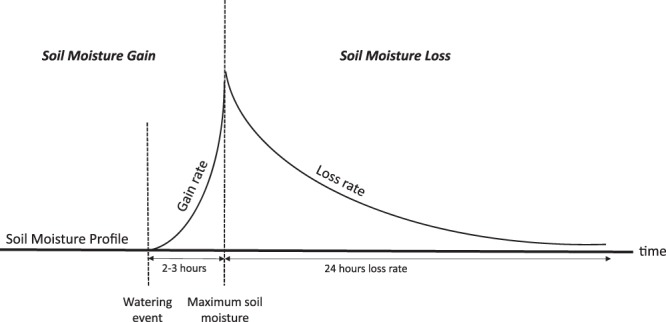
Soil Moisture Gain and Loss profile per watering event: Soil moisture gain rates were calculated based on the time to soil moisture maximums achieved 2–3 hours after each watering event, and soil moisture loss rates were calculated based on the 24 hours after the maximum was achieved. Water holding capacity was significant for both soil moisture gain and loss rates.

Based on data from the individual watering events, a general soil moisture profile was developed and is shown in Fig. 2. The rates of soil moisture gain generally occurred within the first two to three hours after each watering event, with most soils reaching maximum soil moisture levels within 2 hours of the watering event. Thus, soils were highly responsive to watering inputs. Soil moisture loss rates were calculated based on the 24 hours after the maximum soil moisture was achieved; however, some gardeners watered more periodically, and in these cases the soils continued to lose soil moisture until more water was input into the plot.

### Drivers of soil moisture gain and loss within urban gardens

We found that across all categories and the explanatory variables chosen for the two analyses, soil quality management, specifically water holding capacity (%WHC), was the only factor that was significant for both the soil moisture gain and loss rate models (Tables 1 and 2). Greater water holding capacity led to both slower rates of soil moisture gain (LMM: t = −2.36, p = 0.02, Table 1) as well as slower rates of soil moisture loss (LMM: t = −2.42, p = 0.02, Table 2), indicating that high water holding capacity soils provide a more stable soil moisture profile and less fluctuations in soil moisture measurements.

**Table 1:** Linear mixed model (LMM) results for soil moisture gain using plot temperatures (°C), water use (L/m^2^), a vegetation factor (% crop cover), a ground cover factor (% straw cover), and a soil quality factor (% water holding capacity). Analysis of Deviance based on Type II Wald chisquare tests. (Signif. codes: 0 ‘***’ 0.001 ‘**’ 0.01 ‘*’ 0.05). AIC: −659.8, BIC: −632.0; Estimates of variance for random effects: Plot nested within Garden = < 0.001; Garden = < 0.001.

Fixed Effects	Estimate	Std. Error	t-value	Pr(>Chisq)
**Soil Moisture Gain**				
Average Temperature (°C) (24 hours prior)	−1.02 e-03	7.52 e-04	−1.37	0.17
Water Used (L/m^2^)	−3.04 e-05	9.74 e-05	−0.31	0.75
Crop Cover (%)	3.06 e-03	4.18 e-03	0.73	0.46
Straw Cover (%)	1.38 e-04	2.10 e-04	0.66	0.51
Water Holding Capacity (%)	−1.78 e-03	7.52 e-04	−2.36	0.02*

**Table 2:** Linear mixed model (LMM) results for soil moisture loss rates using plot temperatures (°C), water use (L/m^2^), a vegetation factor (% crop cover), a ground cover factor (% straw cover), and a soil quality factor (% water holding capacity). Analysis of Deviance based on Type II Wald chisquare tests. (Signif. codes: 0 ‘***’ 0.001 ‘**’ 0.01 ‘*’ 0.05). AIC: −1534.9, BIC: −1507.9; Estimates of variance for random effects: Plot nested within Garden = < 0.001; Garden = < 0.001.

Fixed Effects	Estimate	Std. Error	t-value	Pr(>Chisq)
**Soil Moisture Loss**				
Average Temperature (°C) (24 hours post)	−1.09 e-05	3.09 e-05	−0.35	0.72
Water Used (L/m^2^)	1.52 e-06	4.04 e-06	0.38	0.71
Crop Cover (%)	2.27 e-04	1.55 e-04	1.47	0.14
Straw Cover (%)	3.45 e-04	1.81 e-04	1.91	0.06
Water Holding Capacity (%)	−6.92 e-05	2.86 e-05	−2.42	0.02 *

**Figure 3:**
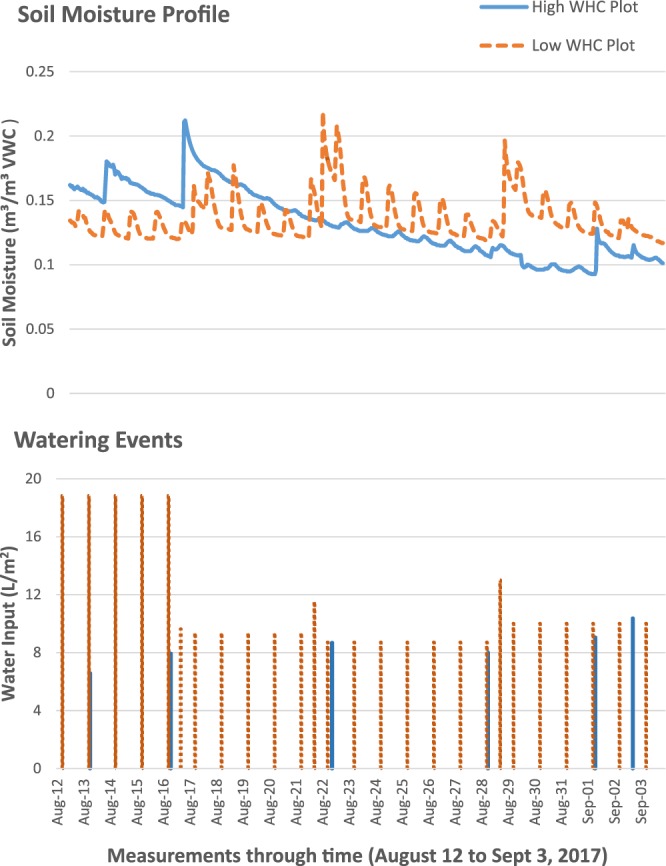
A soil moisture profile from two plots that differ in soil water holding capacity in one garden. The solid line represents a plot with high water holding capacity (40.8% WHC), and the dotted line represents a plot with a low water holding capacity (20.2% WHC) - a two fold difference. Watering events per plot are shown in the bottom figure with solid and dotted line representing high and low water holding capacity plots respectively. The profiles and watering events are from a period from August 12 to September 13, 2017. Note that the low WHC plot received more water, more frequently yet maintains essentially the same trajectory as the soil moisture profile as the high WHC plot.

An example of how water holding capacity can affect soil moisture profiles is shown in Fig. 3. In this figure, the soil moisture profiles of two plots of differing water holding capacity within the same garden are shown for a three week period within the sampling period. One plot has higher water holding capacity (40.8% WHC, solid line) while the other plot has lower water holding capacity (20.2% WHC, dotted line). The two plots follow similar trajectories; however, the plot with lower water holding capacity is watered far more frequently and with greater amounts of water than the plot with higher water holding capacity (Fig. 3). This comparison highlights the role that water holding capacity can have in maintaining soil moisture within the plots.

## Discussion

Urban gardens present a unique socio-ecological system in which individual actors manage crops and other plants in small parcels of land, creating complex microcosms at the local scale^35^. Urban gardeners all make specific management decisions based on their experiences, knowledge, and desires for their own gardening experience^29^. We examined how these practices affect soil moisture retention in individual garden plots. Specifically, we examined two aspects of soil moisture retention at the plot level–soil moisture gain rates and soil moisture loss rates–and how ambient temperature, water use, vegetation, ground cover, and soil quality management affect these gain and loss rates.

Because urban gardening in the California Central Coast is highly reliant on irrigation to maintain adequate amounts of soil moisture, gardeners must make management decisions that increase crop growth while not exceeding garden watering regulations. Thus, it is important to understand which management factors affect the water balance of soils in the plots and how these management factors affect rates of soil moisture gain and loss. The goal of many gardeners and garden managers is to reduce water use, especially during drought periods. In some community gardens, watering is restricted to only two or three days of the week^22^, and soil moisture that can be maintained for more than one day could be useful in protecting those plants from water deficit^36,37^. Because it is difficult to precisely know how much water is ‘enough’ without precision instrumentation, one of the challenges to reduce water use in the gardens is to provide simple strategies that can be widely available to most gardeners. Adaptation methods that can be implemented through gardener management can provide some guidance toward maintaining soil moisture and to conserve water.

In this study, we show that the main management factor that affects the gain and loss rates of soil moisture in the garden plots is soil quality (i.e. water holding capacity). Although plots with higher water holding capacity experienced slower rates of soil moisture gain after a watering event, they also experienced slower rates of soil moisture loss after the event, leading to soils with more stable moisture profiles over time (Tables 1 and 2). This is shown in Fig. 3 where the soil moisture profiles of two plots with different water holding capacities within the same garden are represented. While the two plots follow similar trajectories, the plot with lower water holding capacity received more water and is watered more frequently than the plot with higher water holding capacity. This comparison highlights the role that water holding capacity can have in maintaining soil moisture within the plots, therefore requiring less water and less frequent watering events.

Such results support previous research demonstrating the importance of soil quality to maintain water within the soil^31^. In our garden plots, the rates of gain occurred within the first two to three hours after each watering event, with most soils reaching maximum soil moisture levels within 2 hours of the watering event. The quick response of the soils shows how responsive soils are to watering inputs (Fig. 2), although this occurred at a slower rate in soil with high water holding capacity (Table 1). Water holding capacity was a soil quality variable effective in maintaining more soil moisture in the system post-watering event. This may be because water holding capacity takes into account a variety of soil physical quality measures, such as texture, porosity, and soil organic matter, that are often related to field capacity measurements^38^. The combination of these qualities create a soil system that has the structure and composition to maintain more water in the soil for longer periods of time; thereby allowing for less frequent watering schedule and a smaller amount of water to maintain soil moisture levels.

Our results demonstrate that gardener soil management practices differentially impact water holding capacity and thus their modification could be a key step to improving soil moisture within these gardens. Although water holding capacity is often dependent on soil texture, increasing the organic matter content of soils can increase water holding capacity regardless of texture^39^. Agricultural literature has demonstrated how amendments or organic matter can increase water use efficiency within crop systems^40,41^, with one active area focused on biochar amendments to retain more water from rainfall, increase crop production in non-irrigated dryland regions, and reduce the amount of irrigation water needed to grow crops in irrigated regions^42,43^. Thus, soil management protocols which encourage the use of organic matter amendments, such as compost, in urban gardens could greatly benefit the water holding capacity of the soils, especially for gardens with sandy soils. Although not specifically examined during this study, tillage of soils can also impact on the water holding capacity of agricultural soils, with no-till soils generally exhibiting higher water holding capacity than tilled soils^44,45^. Urban gardeners tend to till and prepare their soils at the beginning of the growing season, but with different levels and types of management. Conservation tillage options that incorporate crop residues within the tillage or use mulch and straw cover to reduce soil surface exposure can help maintain water within the soil systems, while allowing for aeration of the soils^46^.

Additionally, in urban soils, soil aeration and porosity can present a problem because many urban gardens are converted from other land uses such as vacant lots. In these cases, urban soils are often not suitable for planting vegetation because of the high levels of compaction and loss of soil structure and porosity^47,48^. Because of this, gardens often have to re-create and build up the soil within their plots in order to prepare the ground for urban agriculture. This process of creating new soils allows gardeners the opportunity to use compost and other soil amendments to craft potentially better quality soils than that of the original site. Such effects were observed in the soils of the participating gardens within this study, where gardeners in essence created ‘novel’ soils through the use of compost and soil bases purchased or brought in from other sites^29^. The process of creating soils may explain why the estimates of variance for random effects within the linear mixed models (Plot nested within Garden; Garden) were almost indistinguishable from zero, as the re-creation of soil at the individual plot scale potentially reduces the nested effect of the gardens.

### Finding the right balance for urban gardeners

The results of this study will benefit gardeners facing extreme climate effects (such as heat and drought) and water use restrictions by informing plot level management strategies to maintain more stable soil moisture levels for food production. Overall, such results highlight that better soil management and increasing soil quality measures such as water holding capacity are potential solutions for maintaining soil moisture under extreme climate conditions, especially as soils with higher water holding capacity have slower rates of water loss and retain water over a longer period after a watering event. Urban gardeners and garden managers who are concerned about water use and plant health during high heat periods should concentrate on better understanding their soil properties and invest in materials that increase soil quality, such as the use of compost or reducing the compaction of the soil.

Trade-offs in time, effort, and money for the individual management of plots are important considerations for each gardener as developing soils will require both a financial and time investment. However, sharing such information to help gardeners adjust their management decisions and increase water use efficiency may be an essential next step in protecting urban garden food provision and protecting the longevity of these community spaces given reduced water availability. Garden managers may be able to provide soil amendments and free compost as incentives to increase soil quality and reduce water use within gardens. Working with gardeners to understand the motivation behind their decisions, and how they holistically consider water use through their plot management will be an important line of inquiry for helping gardeners manage water efficiently in hot and dry climates.

## Methods

### Plot selection

We selected four urban community gardens across the Central Coast of California. The gardens spanned three counties: Monterey (36.2400° N, 121.3100° W), Santa Clara (37.3600° N, 121.9700° W), and Santa Cruz (37.0300° N, 122.0100° W). Two gardens were selected in Santa Clara County, one garden was selected in Santa Cruz County, and one garden was selected in Monterey County. In each garden, we monitored five individually managed plots, for a total of 20 plots across all four gardens.

Participating gardens and monitored gardener plots were determined from gardener interest in volunteer participation in the study, as they were required to monitor their own water use throughout the period of the study. These gardens were surveyed and monitored for a six-week period from August 5^th^ to September 15^th^, 2017. Gardeners also recorded rainfall using a simple rain gauge installed at the gardens. Although gardeners reported no rainfall during the sampling period, regional climatic data did show low amounts of rain recorded (2 mm in Santa Cruz, California and 1 mm in San Jose, California^49^). In addition to the gardener monitoring of water use within their plots, we examined soil moisture measurements, ambient temperature, vegetation cover, soil quality parameters, and ground cover. Specific methods of data collection are outlined below.

The datasets generated and analysed during the current study are available from the corresponding author on reasonable request. All identifying information of the participants have been removed.

### Ethics approval

All research was conducted under appropriate ethics and protocols in accordance with relevant guidelines and regulations of the gardens. We received an exception from the Institutional Review Board for human subject research through the University of California Santa Cruz (UCSC IRB Protocol #3025; DHHS Office for Human Research Protections, FWA00002797) to conduct the research. Additionally we received consent and permission from the gardens to perform research within their community gardens, and written consent was obtained from each gardener as part of their participation in the project and for the use of each individuals’ water data for the analysis. There is no identifying information for any of the participants in the manuscript or corresponding data files (available upon request).

### Soil moisture measurements

Soil moisture was measured hourly in each of the 20 plots using two soil moisture sensors (m³/m³ Volumetric Water Content (VMC), Decagon EM-5 soil moisture sensors) connected to a data logger (Decagon EM50 Digital Data Logger). Soil moisture sensors were placed within the plot at the depth of 15 to 20 cm in order to measure soil moisture at the primary crop rooting depth. Sensors and data loggers were checked throughout the survey period to ensure that they were in good working order. At the end of the six weeks, the data from the two soil moisture sensors were downloaded, quality checked and cleaned, and averaged. Periods where sensors were disturbed were excluded from the dataset.

We calculated soil moisture gain and loss rates using the averaged soil moisture readings. At each plot, watering data from each gardener was matched to the data so that the soil moisture gain and loss rate of each watering event could be calculated. Soil moisture gain rates were calculated based on the rise of soil moisture over the time period to reach maximum (Fig. 3). Soil moisture loss rates were calculated based on the soil moisture maximum and the soil moisture measurement 24 hours after the maximum was reached (∆ VWC/hour).

### Temperature measurements

Each plot was monitored with a temperature logger (Onset HOBO UA-001-08) to collect hourly ambient temperature measurements over the sample period. Temperature measurements were taken at every plot to account for the potential variability in local or surrounding vegetation structure that may affect temperatures around each plot. Temperature loggers were placed 1.5 meters above the plot at the edge of the plot to record temperatures (°C) directly around the plot. Data loggers were checked and maintained throughout the survey period to ensure that they were in good working order. Data were downloaded at the end of the survey period and quality checked and cleaned.

For each plot, the average temperature 24 hours before and after each watering event were calculated to correspond with the climatic environment at the time of watering gain and loss and to examine if average temperatures affected rates of soil moisture loss.

### Water use data

Water use data was collected with the voluntary participation of 20 community gardeners, adhering to the ethics protocols stated above. Each gardener collected their own water use data over a period of six-weeks so that the watering data could be matched to the recorded soil moisture data. The gardeners were asked to record the number of liters of waters that they used at each watering event throughout the data collection period as well as the time and date of each of these watering events. Each gardener was supplied with a Gardena water monitor attached to their hose to help them measure their water use (Gardena Brand Electronic Garden Hose Water Meters). These were installed directly to each of their faucets at the plot level to ensure that they were only recording their own water use. They were also provided with a clipboard and a data sheet to keep track of the date, time, and the number of liters used for each watering event.

Water use data in the analysis was then averaged by the size of the plot in order to assess the liters of water used per meter squared of gardening space. This was done because garden plots were of slightly differing sizes. Each watering event was considered a separate data point for the analysis. All watering data was cleaned and quality checked against the soil moisture data to ensure that proper dates and times were entered into the gardener data sheets. Watering information that was not clear or confirmed in the soil moisture data were not used in the analysis.

### Vegetation, soil, and ground cover data

Plot level data on vegetation cover, ground cover, and soil characteristics were collected for each plot to assess the types of management used at plot level. Plot size was also collected to account for differences in area for watering.

Vegetation data was collected for each plot to assess the percent cover of crops, weeds, and herbaceous plants. Various types of ground cover, including mulch, straw, rocks, and bare soil were collected in the same manner. This was conducted using a visual assessment and an estimate of the percentage of total plot surface of each type of vegetation and ground cover covering the plot.

Soil data for each plot were collected including two bulk density cores for each plot. Soil samples were collected in the first week of August 2018 when the experiment was set up. We used water holding capacity (%WHC) as the soil quality measure in the analysis as it represents a soil quality factor aligned to maintaining water in the soil and takes into account a number of soil property variables such as soil texture, porosity, and soil organic matter content^38^. To determine soil water holding capacity, we used the bulk density samples following Wilke^50^ (2005) and followed the standardized method that determines the maximum amount of water retained by the soil against gravity by saturating soil samples, draining soils of free water, and evaluating only the water held by the soil. This method uses sieved soils to standardize soil structure, as garden soils are continuously tilled and amended with purchased soil (e.g., potting soil). We filled 2 × 2” cylinders with a perforated base with sieved, fresh soil, and placed them in a water bath overnight. We then capped and placed cylinders on a tray of sand for approximately six hours, allowing soils to drain, and then removed and dried soils (105 °C, 24 hours) to calculate water holding capacity.

### Analysis

We used two linear mixed-effects models (LMMs) to examine the relationship between plot level management factors (explanatory variables) and both soil moisture gain and loss rates (response variables) using maximum likelihood. One model was built for soil moisture gain rate, and another model for soil moisture loss rate as the response with watering event examined as the unit of analysis. We use non-correlated variables, based on a correlation matrix (p < 0.05 used as a threshold) to meet model assumptions^51,52^. The same non-correlated explanatory variables were used for both models: average temperature (24 hours before and after the watering event, °C), water used (per watering event, L/m^2^ of plot surface); crop cover (%, square root transformed); straw cover (%); and WHC (%) (Table 3). Explanatory variables were transformed to meet assumptions of normality of the linear model^51,52^.

**Table 3:** Plot level information was collected across five categories to describe plot conditions. Only one non-correlated explanatory variable was chosen from each category for the soils moisture gain and loss rate analyses. The same variables were used for both models.

Plot Level Information – Categories for analysis	Representative explanatory variables used in the analysis
Ambient Temperature	Average Temperature (°C) (24 hours pre and post watering event)
Water Use	Water Used (L/m^2^)
Vegetation	Crop Cover (%)
Ground Cover	Straw Cover (%)
Soil Quality	Water Holding Capacity (%)

The LMMs modelled plot scale measurements and included plot nested within garden as a random effect to account for pseudo-replication and multiple measurements within each plot^53^. We used both the *lme* function in the *lme4* package^54^ as well as the *Anova* function in the *car* package^55^ in the R statistical environment^56^ to perform the analysis. Analysis of Deviance Tables were created using Type II Wald chi-square tests based on the *car* package.
